# Assessment of Serum Sestrin 2 Levels in Women With Polycystic Ovary Syndrome: A Single-Center Cross-Sectional Case-Control Study

**DOI:** 10.7759/cureus.80440

**Published:** 2025-03-11

**Authors:** Lohit Kumbar, Pramila Kalra, Hanumantha Rao Maddukuri

**Affiliations:** 1 Endocrinology and Diabetes, Shri Dharmasthala Manjunatheshwara (SDM) College of Medical Sciences and Hospital, Dharwad, IND; 2 Endocrinology, Mathikere Sampangi (MS) Ramaiah Medical College, Bengaluru, IND; 3 Endocrinology and Metabolism, Mathikere Sampangi (MS) Ramaiah Medical College, Bengaluru, IND

**Keywords:** hormonal imbalance, metabolic syndrome, polycystic ovary syndrome, roc curve analysis, serum biomarker, sestrin 2

## Abstract

Objective

Sestrin 2 (SESN2) is a stress-responsive protein implicated in metabolic regulation and oxidative stress. This study aims to assess serum SESN2 levels in treatment-naive subjects with polycystic ovary syndrome (PCOS) and compare them to healthy controls.

Methods

The single-center cross-sectional case-control study was conducted from February 2020 to February 2021 at a multispecialty center in Bengaluru, India. The study included 37 newly diagnosed, drug-naive women with PCOS and 46 healthy, age-matched and body mass index (BMI)-matched controls. SESN2 levels, along with anthropometric and metabolic parameters, were measured for both groups. Ethical approval was obtained, and all participants provided informed consent.

Results

The study involved 83 participants, divided into two groups: 37 patients with PCOS and 46 healthy controls. Significant differences were observed between the groups in terms of weight, hip circumference, and serum SESN2 levels. The PCOS group had higher weight (P=0.024) and larger hip circumference (P=0.037), while the control group exhibited a higher waist-to-hip ratio (WHR) (P=0.007). Although no statistically significant difference was found in body mass index (BMI), the PCOS group showed elevated serum SESN2 levels (P=0.024). Correlation analysis revealed a negative correlation between follicle-stimulating hormone (FSH) and SESN2 levels in the PCOS group. Receiver operating characteristic (ROC) curve analysis determined a SESN2 cutoff of 1.484 ng/mL, which demonstrated moderate sensitivity and specificity for distinguishing PCOS patients from healthy controls.

Conclusion

SESN2 levels were significantly higher in PCOS patients, with moderate correlations observed with metabolic and anthropometric parameters. SESN2 may play a role in the hormonal and metabolic disturbances characteristic of PCOS, highlighting its potential as a biomarker for PCOS diagnosis and management.

## Introduction

The global prevalence of polycystic ovary syndrome (PCOS), a prevalent endocrine disorder that affects women during adolescence and reproductive years, ranges from 4% to 21%, while in India, it varies between 2% and 35% [1,2]. According to the Rotterdam criteria (2003), the diagnosis of PCOS is established when at least two of the following three features are present: hyperandrogenism, polycystic ovarian morphology on ultrasound, and menstrual irregularities [3]. PCOS is closely linked with metabolic disorders, and its development and persistence are heavily influenced by insulin resistance (IR). Newer markers are warranted to more accurately assess IR. Research has identified several surrogate markers to assist in the determination of IR, including novel proteins such as copeptin, irisin, plasminogen activator inhibitor-1 (PAI-1), zonulin, adipocytokines (such as adiponectin, visfatin, vaspin, and apelin), kisspeptin, resistin, leptin, retinol-binding protein 4 (RBP4), and sestrins (SESNs). However, the role of these markers remains uncertain [4].

SESNs are highly conserved proteins with a variety of biological functions. Under stress conditions such as DNA damage, hypoxia, starvation, growth factor depletion, radiation, and oxidative stress, SESNs are upregulated in cells. These proteins help protect cells from oxidative and genotoxic stress, earning them the name stress-inducible metabolic regulators. The three distinct types of SESN proteins, namely, sestrin 1 (SESN1), sestrin 2 (SESN2), and SESN, share approximately 50% identical amino acid sequences. SESN2, also referred to as hypoxia-inducible gene 95 (Hi95) due to its induction under hypoxic conditions, can also be activated by oxidative stress and DNA damage [5]. SESN2 exerts its effects through the p53 pathway. Furthermore, SESN is associated with IR, and its role in autophagy helps protect insulin sensitivity and regulate glucose metabolism. Emerging evidence suggests that SESN2 plays a pivotal role in reducing IR by regulating glucose and lipid homeostasis [5].

In women with PCOS, the dysregulation of autophagy has been observed in the endometrium and ovarian granulosa cells and is regulated by various proteins, including SESN2. Two primary mechanisms have been identified for SESN2's protective function: first, SESN2 acts as an antioxidant, reducing excessive reactive oxygen species. Second, p53-induced SESN2 activates adenosine monophosphate-activated protein kinase (AMPK), which inhibits the mammalian target of rapamycin complex 1 (mTORC1) and triggers autophagy under stressful conditions [6].

Previous studies have shown that the deficiency of SESN2 is associated with IR and abnormal glucose and lipid metabolism, likely due to the downregulation of AMPK and the overactivation of mTORC1 [7]. Furthermore, SESN2 levels have been reported to be lower in obese individuals at an early age [8]. The current study aims to assess serum SESN2 levels in treatment-naive women with PCOS and compare them to healthy controls, exploring its potential as a stress-responsive protein and a biomarker in the pathophysiology of this metabolic syndrome.

## Materials and methods

The single-center cross-sectional case-control study included patients who visited the outpatient department of the department of endocrinology at a multispecialty hospital in Bengaluru from February 2020 to February 2021. The inclusion criteria for the study were drug-naive PCOS patients aged 18-45 years who met the Rotterdam criteria, had not received treatment in the past three months, and were not planning pregnancy within the next three months [9]. Exclusion criteria included pregnant or lactating women, those using oral contraceptive pills, individuals with chronic inflammatory or autoimmune diseases, and patients with conditions such as diabetes mellitus, hypertension, renal failure, thyroid disorders, acute infections, or other serious illnesses. Additionally, women with a history of current or past malignancy or acute or chronic liver disease were excluded from the study.

The participants were randomly assigned, with the case group consisting of women with PCOS and the control group comprising healthy volunteers. SESN2 levels were measured for both groups at baseline. All participants provided written informed consent, and the study was approved by the Institutional Ethics Committee of Mathikere Sampangi (MS) Ramaiah Medical College (approval number: MSRMC/EC/PG-11/03-2019).

Sample size calculation

Based on the study conducted by Chung et al., the SESN2 levels noted in the non-metabolic syndrome group were 5.08 (4.15, 6.53) ng/mL, while in the metabolic syndrome group, they were 5.61 (4.46, 6.80) ng/mL, with a P-value of 0.052 [10]. In the present study, expecting an effect size of 0.8, with a power of 85% and an alpha error of 5%, the sample size was calculated to be 24 in each group.

Data collection

Anthropometric measurements, including body weight, height, body mass index (BMI), waist circumference, hip circumference, and waist-to-hip ratio (WHR), as well as fasting blood samples for insulin and SESN2, were collected from all study participants after an overnight fast of at least eight hours. Additional metabolic parameters were assessed in the case group to explore the potential influence of metabolic factors, including serum levels of fasting glucose, total cholesterol, triglycerides, high-density lipoprotein (HDL), low-density lipoprotein (LDL) cholesterol, and other relevant metabolic markers. These parameters were not used for baseline comparisons between the two groups but were analyzed to better understand metabolic abnormalities associated with PCOS. Insulin levels were measured using chemiluminescent immunoassay (CLIA), and insulin resistance (IR) was calculated using the homeostasis model assessment of IR (HOMA-IR). Serum SESN2 levels were determined using an enzyme-linked immunosorbent assay (ELISA) kit (Bioassay Technology Laboratory, Shanghai, China), following the manufacturer's protocol.

Statistical analysis

Statistical analyses were performed using the Statistical Package for Social Sciences (SPSS) version 21.0 (IBM Corp., Armonk, NY). Normally distributed data were presented as mean±SD, and nonparametric data were logarithmically transformed. Comparisons between the case group and the control group were conducted using Student's t-test for normally distributed variables or the Mann-Whitney U test for non-normally distributed variables. Correlations between SESN2 levels and various metabolic and anthropometric parameters were assessed using the Pearson or Spearman correlation analysis, depending on the distribution of the data.

Receiver operating characteristic (ROC) curve analysis was used to assess the potential of SESN2 as a marker for distinguishing PCOS patients from healthy controls. The cutoff value for SESN2 was set at 1.484 ng/mL, determined based on the 75th percentile of serum SESN2 levels in the control group. Multivariate regression analysis was performed to identify independent predictors of SESN2 levels. A P-value of <0.05 was considered statistically significant.

## Results

Comparison of baseline characteristics

A total of 83 participants were included, comprising 37 women aged 18-45 years diagnosed with PCOS based on the Rotterdam criteria, forming the case group [9]. The control group consisted of 46 age- and BMI-matched healthy women without any clinical signs of PCOS.

Increased weight (P=0.024) and hip measurements (P=0.037) were noted in the case group compared to the control group. However, the waist-to-hip ratio (WHR, P=0.007) was significantly higher in the control group. Additionally, women in the case group had significantly higher levels of SESN2 (P=0.024). However, the difference in BMI was not statistically significant between the groups (P=0.07). No significant differences were found in BMI, age, height, waist circumference, and serum insulin levels between the groups (P>0.05) (Table 1).

**Table 1:** Comparison of baseline characteristics between the case group and healthy controls Test statistics include t-values for independent t-tests and U-values for Mann-Whitney U tests wherever applicable WHR, waist-to-hip ratio; BMI, body mass index; SESN2, sestrin 2

Parameters	Case (n=37)	Control (n=46)	P-value	Test statistic value
Age (years)	24.73+3.47	25.17+3.99	0.595	t=-0.53
Weight (kg)	67.63+16.54	60.74+8.01	0.024	U=1246
Height (cm)	158.05+6.29	156.2+4.55	0.122	t=1.56
Hip circumference (cm)	89.04+16.19	82.89+7.12	0.037	U=538
Waist circumference (cm)	80.13+14.99	76.74+7.03	0.211	t=1.27
WHR	0.90+0.05	0.93+0.03	0.007	U=532
BMI (kg/m²)	26.97+5.92	24.94+3.46	0.07	t=1.85
Serum insulin (µU/mL)	18.47+17.65	13.48+15.08	0.169	t=1.39
Serum SESN2 (ng/mL)	1.665+0.671	1.348+0.549	0.024	U=1065

Metabolic parameters evaluated in the test group

The glycemic profile indicated normoglycemia with fasting blood sugar levels at 91.16±8.98 mg/dL and glycosylated hemoglobin (HbA1c) at 5.57%±0.39%. Renal function was preserved with creatinine levels of 0.68±0.15 mg/dL, and thyroid function appeared normal with thyroid-stimulating hormone (TSH) levels at 2.50±1.41 µIU/mL. The hormonal profile showed elevated prolactin levels (19.41±10.79 ng/mL), while follicle-stimulating hormone (FSH) (5.28±1.41 mIU/mL) and luteinizing hormone (LH) (8.65±5.25 mIU/mL) were within acceptable ranges. Testosterone levels (0.39±0.14 ng/dL) were lower than expected, suggesting potential hormonal imbalances. The lipid profile revealed borderline high total cholesterol (187±32 mg/dL) and LDL (100.22±25.51 mg/dL) levels, with reduced HDL (37.5±4.5 mg/dL) and elevated triglycerides (161.4±57.9 mg/dL), indicating dyslipidemia (Table 2).

**Table 2:** Findings of metabolic parameters noted in the case group FBS, fasting blood sugar; TSH, thyroid-stimulating hormone; PRL, prolactin; FSH, follicle-stimulating hormone; LH, luteinizing hormone; LDL, low-density lipoprotein; HDL, high-density lipoprotein; HbA1c, glycosylated hemoglobin

Parameters	Mean SD (n=37)
FBS (mg/dL)	91.16+8.98
HbA1c (%)	5.57+0.39
Creatinine (mg/dL)	0.68+0.15
TSH (µIU/mL)	2.50+1.41
PRL (ng/mL)	19.41+10.79
FSH (mIU/mL)	5.28+1.41
LH (mIU/mL)	8.65+5.25
Testosterone (ng/dL)	0.39+0.14
Total cholesterol (mg/dL)	187+32
LDL (mg/dL)	100.22+25.51
HDL (mg/dL)	37.5+4.5
Triglycerides (mg/dL)	161.4+57.9

ROC analysis

**Figure 1:**
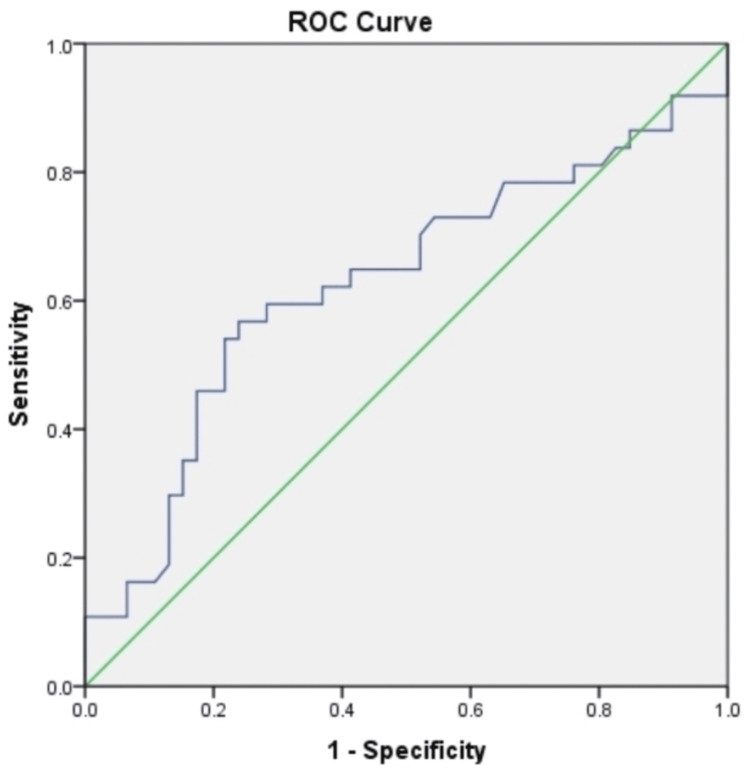
ROC curve analysis for discriminating against patients with PCOS from healthy controls ROC: receiver operating characteristic

The receiver operating characteristic (ROC) analysis indicated that a cutoff value of 1.484 ng/mL for SESN2 provided a sensitivity of 56.7% and a specificity of 76.08% in differentiating PCOS patients from healthy controls, with an area under the curve (AUC) of 0.63 (Figure 1).

Correlation analysis

The correlation between various metabolic and anthropometric parameters and SESN2 levels demonstrated that FSH had a moderate negative correlation with SESN2 (r=-0.333), which was statistically significant (P=0.044). LH (r=0.308) showed a moderate positive correlation, with a p-value approaching significance (P=0.064), while serum insulin (r=0.331) exhibited a very weak positive correlation, also nearing statistical significance (P=0.084). Weight, height, waist circumference, hip circumference, BMI, creatinine, TSH, and prolactin all showed weak positive correlations with SESN2, but these correlations were not statistically significant. Waist-to-hip ratio, testosterone, and LDL also exhibited very weak positive correlations with SESN2, which were not statistically significant. Both age and waist-to-hip ratio revealed weak negative correlations with SESN2, but these too were not statistically significant (Table 3).

**Table 3:** Correlation analysis of serum SESN2 levels with various metabolic and anthropometric parameters BMI, body mass index; WHR, waist-to-hip ratio; FBS, fasting blood sugar; TSH, thyroid-stimulating hormone; PLR, prolactin; FSH, follicle-stimulating hormone; LH, luteinizing hormone; LDL, low-density lipoprotein; Hba1c, glycosylated hemoglobin

Variables	Sestrin 2 (SESN2) (r)	P-value
Age (years)	-0.134	0.226
Weight (kg)	0.165	0.136
Height (cm)	0.173	0.118
Hip circumference (cm)	0.065	0.559
Waist circumference (cm)	0.061	0.584
BMI (kg/m²)	0.110	0.324
WHR	-0.038	0.733
FBS (mg/dL)	-0.039	0.817
Hba1c (%)	-0.088	0.606
Creatinine (mg/dL)	0.226	0.184
TSH (µIU/mL)	0.077	0.650
PRL (ng/mL)	0.150	0.377
FSH (mIU/mL)	-0.333	0.044
LH (mIU/mL)	0.308	0.064
Testosterone (ng/dL)	-0.082	0.628
LDL (mg/dL)	0.103	0.545
Serum insulin (µU/mL)	0.331	0.084

In the regression analysis conducted for PCOS cases, the model explained 23.7% of the variance in sestrin 2 levels (R²=0.237), though the overall model was not statistically significant (P=0.514). Among the independent variables, height² (P=0.092), height (P=0.105), and weight (P=0.127) showed a trend toward significance, suggesting a possible association with sestrin 2 levels, though not at the conventional significance threshold. Other variables, including age, BMI, waist circumference, waist-to-hip ratio, insulin, and hip circumference, did not show significant relationships with sestrin 2.

## Discussion

This study explored SESN2 as a potential marker for distinguishing women with PCOS from healthy controls, with findings suggesting a significant difference in SESN2 levels between the two groups. The increased SESN2 levels in the PCOS group were associated with various metabolic disturbances such as dyslipidemia (low HDL, high LDL, and elevated triglycerides), insulin resistance, and a higher waist-to-hip ratio, which are key risk factors for cardiovascular diseases and metabolic syndrome in PCOS patients.

The present study found that the PCOS group had a significantly higher average weight compared to the control group. This finding aligns with the research of Zhang et al., who reported that women with PCOS are more likely to exhibit obesity and have a higher weight [11]. Similarly, Mahabady et al. observed that the mean weight and BMI in individuals with PCOS were significantly higher than in the control group [12]. Atakul et al. also noted that the average weight of patients in the PCOS group was higher than that of healthy controls [13]. Additionally, Teede et al. documented higher weight in women with PCOS across all time points, including the first survey [14]. Jurczewska et al. reported significantly higher body weight levels in PCOS patients, further supporting the association between PCOS and increased body weight [15].

The current study found that hip circumference was significantly greater in the PCOS group compared to the control group, a result that aligns with previous research. Chitme et al. reported a mean hip circumference of 109.22±17.39 in PCOS patients, significantly higher than that of women in the control group [16]. Similarly, Mohapatra and Samantaray observed a significantly increased hip circumference in individuals with PCOS, with a mean of 101.47±9.320 [1].

Additionally, the study revealed that the WHR was significantly lower in the PCOS group compared to the control group, suggesting potential differences in fat distribution. This finding aligns with Zhang et al., who observed that the PCOS group had a WHR of 0.86±0.07, significantly higher than the 0.81±0.07 in the control group [11]. Similarly, Shirazi et al. found increased WHR in individuals with PCOS [17]. These findings reinforce the association between PCOS and increased hip circumference, as well as a more central fat distribution, highlighting the importance of considering fat distribution as a key metabolic characteristic in PCOS.

The present study found significantly higher serum SESN2 levels in the PCOS group compared to the control group. This study specifically focused on newly diagnosed, drug-naive PCOS patients, effectively ruling out the potential influence of medications on SESN2 levels. This finding contrasts with some previous literature, such as a study by Saeedi et al., which reported significantly lower plasma SESN2 levels in PCOS patients compared to healthy individuals [5]. Similarly, Nourbakhsh et al. found that SESN2 concentrations were significantly lower in obese children compared to their normal-weight counterparts [8]. In another study by Çatal and Kovalak, serum SESN2 levels were significantly lower in the obese PCOS group compared to both the control and non-obese PCOS groups (P=0.001 and P=0.0001), with the non-obese PCOS group also showing significantly lower SESN2 levels compared to the control group (P=0.0001) [18].

In contrast, Wang et al. [19] and Rai and Dey [20] observed an increase in circulating SESN2 levels in patients with cardiovascular and pulmonary diseases, cancer, and neurodegenerative disorders. Additionally, Wang et al. demonstrated that high plasma concentrations of SESN2 in patients with chronic heart failure were associated with a higher occurrence of major adverse cardiac events and predicted poorer outcomes [21]. Chai et al. [22] and Kang et al. [23] reported considerably high levels of plasma SESN2 in patients with obstructive sleep apnea and asthma, suggesting a compensatory response to chronic hypoxia. Furthermore, studies by Ro et al. [24] and Tsilioni et al. [25] found elevated plasma SESN2 levels in patients with malignancies such as lung and colorectal cancers, which may indicate a tumor-suppressive role for the protein. Rai et al. [26] and Kamalzadeh et al. [27] also showed significantly increased serum SESN2 levels in patients with Parkinson's disease and Alzheimer's disease, with a significant negative correlation to mental and cognitive function.

In the current study, a negative correlation was observed between FSH and SESN2 levels in PCOS patients, suggesting that as FSH levels increase, SESN2 levels tend to decrease or vice versa. Women with PCOS exhibited significantly higher levels of FSH (4.38±2.05) [28]. However, no correlation was found between serum sestrin levels and other hormones such as FSH, LH, prolactin, testosterone, dehydroepiandrosterone sulfate (DHEAS), and estradiol. On the other hand, a positive correlation was identified between the LH/FSH ratio and sestrin levels [29].

Regarding diagnostic potential, the current ROC analysis showed that a SESN2 cutoff value of 1.484 ng/mL yielded a sensitivity of 56.7% and a specificity of 76.08% for distinguishing PCOS patients from healthy controls, with an AUC of 0.63 (95% confidence interval). This finding contrasts with Saeedi et al., who reported significantly lower plasma SESN2 levels in PCOS patients compared to healthy subjects. Their study identified a cutoff value of 420.5 ng/L, demonstrating a higher sensitivity (83.87%) but lower specificity (46.88%) for distinguishing individuals with and without PCOS. Additionally, SESN2 levels significantly contributed to their model, with an odds ratio of 0.995, indicating that higher SESN2 levels were associated with a lower likelihood of PCOS [5]. In a similar context, Bestel et al. conducted ROC analysis and found that serum SESN2 levels were strong indicators for diagnosing PCOS, with an impressive AUC of 99.4% at a cutoff value of 4.69 ng/mL (P<0.001; 95% CI: 96.4%-100%; sensitivity: 100%; specificity: 96.7%) [29].

Strengths, limitations, and future directions

This study provides valuable insights into the relationship between metabolic parameters and SESN2 levels in women with PCOS, utilizing a cross-sectional case-control design to ensure temporal accuracy. The inclusion of age- and BMI-matched healthy controls, along with newly diagnosed, drug-naive women with PCOS, strengthens the validity of the comparisons between the two groups. The comprehensive measurement of metabolic parameters, including FBS, HbA1c, insulin, and cholesterol levels, offers a thorough understanding of the metabolic profile in PCOS patients. Advanced statistical methods, such as correlation analysis and ROC curve analysis, enhance the robustness of the findings and their clinical relevance. However, the single-center design may limit the generalizability of the results, and a multicenter study could have provided more diverse data. The small sample size, while adequate for effect size calculations, may reduce statistical power to detect subtle differences. The study primarily presents cross-sectional data, and longitudinal studies would offer a better understanding of changes in SESN2 levels and metabolic parameters over time. Additionally, the exclusion of certain comorbidities limits the broader applicability of the findings. Additional studies with larger sample sizes and more diverse populations are needed to validate SESN2 as a reliable diagnostic and prognostic marker for PCOS, particularly in relation to its role in predicting metabolic complications and cardiovascular risks.

## Conclusions

The study highlights the potential role of SESN2 as a biomarker for differentiating PCOS patients from healthy controls. The moderate correlations observed with metabolic and anthropometric parameters suggest a possible link between SESN2 and hormonal dysregulation in PCOS. The findings point to the need for further exploration to confirm the diagnostic and clinical relevance of SESN2 in managing PCOS and its associated metabolic abnormalities.
